# Electrical Features of the Diabetic Myocardium. Arrhythmic and Cardiovascular Safety Considerations in Diabetes

**DOI:** 10.3389/fphar.2021.687256

**Published:** 2021-07-08

**Authors:** Mónica Gallego, Julián Zayas-Arrabal, Amaia Alquiza, Beatriz Apellaniz, Oscar Casis

**Affiliations:** Department of Physiology, Faculty of Pharmacy, University of the Basque Country UPV/EHU, Vitoria-Gasteiz, Spain

**Keywords:** ion channels, currents, cardiac, arrhythmia, antidiabetics, CVOT, TQT

## Abstract

Diabetes is a chronic metabolic disease characterized by hyperglycemia in the absence of treatment. Among the diabetes-associated complications, cardiovascular disease is the major cause of mortality and morbidity in diabetic patients. Diabetes causes a complex myocardial dysfunction, referred as diabetic cardiomyopathy, which even in the absence of other cardiac risk factors results in abnormal diastolic and systolic function. Besides mechanical abnormalities, altered electrical function is another major feature of the diabetic myocardium. Both type 1 and type 2 diabetic patients often show cardiac electrical remodeling, mainly a prolonged ventricular repolarization visible in the electrocardiogram as a lengthening of the QT interval duration. The underlying mechanisms at the cellular level involve alterations on the expression and activity of several cardiac ion channels and their associated regulatory proteins. Consequent changes in sodium, calcium and potassium currents collectively lead to a delay in repolarization that can increase the risk of developing life-threatening ventricular arrhythmias and sudden death. QT duration correlates strongly with the risk of developing *torsade de pointes*, a form of ventricular tachycardia that can degenerate into ventricular fibrillation. Therefore, QT prolongation is a qualitative marker of proarrhythmic risk, and analysis of ventricular repolarization is therefore required for the approval of new drugs. To that end, the Thorough QT/QTc analysis evaluates QT interval prolongation to assess potential proarrhythmic effects. In addition, since diabetic patients have a higher risk to die from cardiovascular causes than individuals without diabetes, cardiovascular safety of the new antidiabetic drugs must be carefully evaluated in type 2 diabetic patients. These cardiovascular outcome trials reveal that some glucose-lowering drugs actually reduce cardiovascular risk. The mechanism of cardioprotection might involve a reduction of the risk of developing arrhythmia.

## Introduction

Diabetes affected 422 million adults in 2014 (WHO, 2021), but the incidence and the associated socio-sanitary cost are steadily rising. Diabetes is a chronic metabolic disease characterized by hyperglycemia in the absence of treatment. Type 1 diabetes (T1D), caused by insufficient or no insulin production, is the less common form. Type 2 diabetes (T2D) accounts for more than 90% of the cases and starts with insulin resistance, but later progresses towards various degrees of *β*-cell dysfunction. As a result, many type 2 diabetic patients may need exogenous insulin.

In the last decades, available pharmacologic approaches have significantly improved the life span and the quality of life of diabetic patients. However, over time, diabetes-associated complications have emerged. Among them, cardiovascular disease stands out because is the major cause of mortality and morbidity in diabetic patients. In fact, the Framingham study reported that patients with type 2 diabetes were twice as likely as healthy people to die from cardiovascular diseases (Kannel et al., 1974). Currently, due to the advances in diabetes management, many diabetic patients achieve good glycemic control. However, cardiovascular complications remain (Food and Drug Administration, 2008; Duckworth et al., 2009) and are still the leading cause of death (Preis et al., 2009; Xu and Rajaratnam, 2017).

Besides inducing cardiovascular disease, diabetes changes the myocardial structure leading to a cardiac dysfunction known as diabetic cardiomyopathy. Diabetic cardiomyopathy was first described by Rubler et al., in 1972 as heart failure in diabetics without hypertension, myocardial ischemia, congenital or valvular disease (Rubler et al., 1972). It is currently defined as a ventricular dysfunction in the absence of coronary artery disease or hypertension (Bugger and Abel, 2014). In fact, the Framingham study described the association between diabetes and cardiac hypertrophy independently from blood pressure (Kannel et al., 1974). Moreover, the Cardiovascular Health study (Lee et al., 1997) and the Strong Heart study (Devereux et al., 2000) confirmed the association between diabetes and increased left ventricular mass and wall thickness with compromised diastolic and systolic function. Different mechanisms have been proposed to promote the development of diabetic cardiomyopathy and damage the heart. These include cardiac insulin resistance; metabolic remodeling with abnormal free fatty acids metabolism and lipotoxicity; mitochondrial dysfunction with increased ROS production; accumulation of advanced glycation end products and collagen; abnormalities in calcium handling; pro-inflammatory responses; activation of the renin-angiotensin-aldosterone system and autonomic neuropathy with increased sympathetic activity (reviewed in Casis and Echevarria, 2004; Battiprolu et al., 2013; Bugger and Abel, 2014; Onay-Besikci, 2014; Grisanti, 2018; Jia et al., 2018; Palomer et al., 2018; Quagliariello et al., 2020).

*Torsade de pointes* (TdP) is a ventricular arrhythmia characterized by a change in the amplitude and a twisting of the QRS complexes around the isoelectric line in the electrocardiogram. TdP usually ends spontaneously, but in some cases may degenerate into lethal ventricular fibrillation. Long QT syndrome is an inherited or drug-induced arrhythmia syndrome characterized by a prolongation of the QT interval (Sanguinetti and Tristani-Firouzi, 2006; Schwartz et al., 2012) that causes *torsade de pointes*, ventricular fibrillation and sudden death. Since TdP occurs in the setting of prolonged QT intervals, QT duration and heart rate-corrected QT duration (QTc) have become qualitative markers of proarrhythmic risk. The electrocardiogram of both type 1 and type 2 diabetic patients often shows prolonged QT (Bellavere et al., 1988; Chambers et al., 1990; Jermendy et al., 1990; Ewing et al., 1991; Veglio et al., 2000; Brown et al., 2001; Ninkovic et al., 2016), which increases the risk of ventricular arrhythmia (Tse et al., 2016; Hegyi et al., 2019). At the cellular level, diabetes lengthens the cardiac action potential duration due to changes in the expression and electrophysiological properties of various ion channels (Lengyel et al., 2007; Torres-Jacome et al., 2013; Gallego and Casis, 2014).

In addition, arrhythmogenesis in diabetes might be amplified by other factors like autonomic dysregulation (Jermendy, 2003; Spallone et al., 2011; Chen et al., 2019), inflammation (Karam et al., 2017) and the presence of comorbidities such as hypertension (Yiu and Tse, 2008). This review focuses on the electrical characteristics of the diabetic myocardium, such as reduced conduction velocity and prolonged ventricular repolarization. Since these alterations may increase the risk of arrhythmia, evaluating the proarrhythmic risk of new drugs is very relevant for the case of glucose-lowering drugs.

Since 2005, potential proarrhythmic risk must be evaluated for the approval of any new drug, including new anti-hyperglycemic drugs. Currently, the Thorough QT/QTc (TQT) studies are performed with premarketing drugs in order to exclude those that prolong the QT interval (ICH E14). At the same time, concerns about cardiovascular risk in diabetic patients has guided the policy of the regulatory agencies to establish the safety of new glucose-lowering drugs. Therefore, cardiovascular outcome trials (CVOTs) that evaluate specific cardiovascular endpoints are being conducted on type 2 diabetic patients (Goldfine, 2008). Furthermore, the information is being incorporated into the standards of medical care (Scheen, 2020).

**FIGURE 1:**
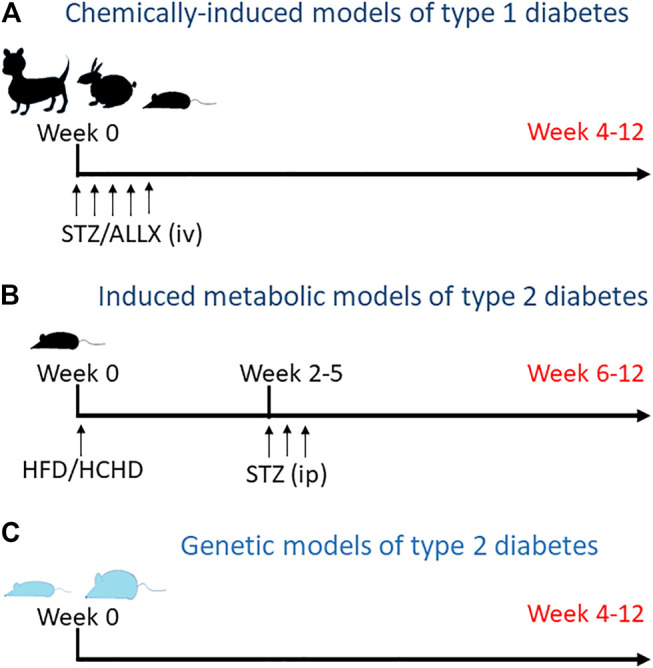
Animal models for diabetes. **(A)** Type 1 diabetes is induced by intravenous injection of streptozotocin or alloxane to kill pancreatic beta cells. Depending on the species, one or more consecutive injections of the toxic are required. **(B)** Metabolic models for type 2 diabetes include diet-induced insulin resistance followed by mild to medium pancreatic toxicity. Animals are fed with high fat and/or high carbohidrate diet (HFD and HCHD, respectively) for some weeks to generate insulin resistance and then receive one or more intraperitoneal injections of low dose streptozotocin to reduce beta cell mass. **(C)** Genetically hyperinsulinemic and/or hyperglycemic animals, lean and obese, spontaneously develop diabetes over time.

In the preclinical setting, animal models, mainly rodents, but also guinea pigs, rabbits and dogs, are commonly used for the study of the diabetic heart. Currently, there are many different animal models for T1D and T2D (Figure 1). These have been extensively reviewed, and include spontaneous, transgenic as well as surgically, chemically and diet-induced models (Masiello, 2006; King, 2012; Al-awar et al., 2016; King and Bowe, 2016). Most of our knowledge regarding cardiac electrical remodeling in diabetes derives from studies with animal models of T1D (Magyar et al., 1992; Shimoni et al., 1994; Casis et al., 2000; Lengyel et al., 2007; Lengyel et al., 2008). Streptozotocin (STZ) and alloxane are glucose analogues that destroy pancreatic *β*-cells (Lenzen, 2008) and have been, for decades, the choice for inducing experimental diabetes because the procedure is simple and reliable. T2D has a different origin and pathophysiology. In humans, it starts with insulin resistance and eventually there is a loss of functional *β*-cells and hyperglycemia. In order to recapitulate this situation, a number of animal models have been generated using different strategies. Among them are the leptin-receptor deficient obese mice (Lepr^db/db^), the non-obese Goto-Kakizaki (GK) rat with defective *β*-cells, the obese Otsuka Long Evans Tokushima Fat rat (OLETF) or the Zucker diabetic fatty rats (ZDF). In addition, metabolic models that combine high fat and/or high-carbohydrate diet with an intraperitoneal low-dose of STZ (Ionut et al., 2010; Podell et al., 2017) are becoming popular. Progressive feeding on high caloric food leads to glucose intolerance and insulin resistance, whereas STZ provides the loss of functional beta cell mass required establishing diabetes.

In the following section, we will discuss the electrical characteristics of the diabetic myocardium that may eventually increase the risk of arrhythmia: from changes in the electrocardiogram and the action potential, to altered behavior of sodium, calcium and potassium channels. Then, we will examine the proarrhythmic safety of antidiabetic drugs and the recommended treatments for type 2 diabetic patients at cardiovascular risk.

## Cardiac Electrical Remodeling

### Alterations in the Cardiac Conduction System in Diabetes

Although diabetic patients frequently have increased heart rate (Ziegler et al., 2008), diagnosis of bradyarrhythmia and the need for pacemaker treatment are also more frequent in type 2 diabetic patients than in control subjects (Rautio et al., 2020). Among the diabetes-induced electrical disturbances, there is a nodal dysfunction that may be related to autonomic dysregulation. In line with this, leptin-receptor deficient db/db mice showed reduced sinus node recovery time and relative autonomic denervation that increased the risk of developing arrhythmia (Soltysinska et al., 2014). On the other hand, there are intrinsic abnormalities in the sinoatrial node that affect the generation and conduction of electricity. For example, the non-obese type 2 diabetic Goto-Kakizaki (GK) rats had a reduced expression of pacemaker channels and connexins. Among others, genes encoding connexins Cx40, Cx43, and Cx45, as well as the hyperpolarization-activated cyclic nucleotide-gated channel 4 (HCN4) that drives the funny current I_f_, were downregulated (Howarth et al., 2018).

Some diabetic patients display slow ventricular depolarization that is visible in the electrocardiogram as an increase in QRS duration (Singleton et al., 2020). Reduced expression of HCN4, connexins and ion channels has also been confirmed in different regions of the cardiac conduction system in the STZ models of T1D (Howarth et al., 2007; Watanabe et al., 2012; Zhang et al., 2019).

Fibroblasts are the most abundant non-cardiomyocyte cells in atria and ventricles. Cardiac fibroblasts can undergo an activation process and differentiate to myofibroblasts that express connexins Cx43 and Cx45. Through these gap junction proteins, myofibroblasts directly interact with cardiomyocytes modulating their electrophysiological behavior and reducing cardiac impulse conduction (Miragoli et al., 2006). Diabetes favors the phenotype switch to myofibroblasts in the heart. Thus, differentiation to myofibroblasts is increased in hearts from STZ-treated and Zucker diabetic rats, which are animal models of T1D and T2D, respectively (Li et al., 2012a; Fowlkes et al., 2013).

Prior to the development of fibrosis, diabetes can induce a lateralization of connexin43 proteins that may impair electrical coupling. Although normal baseline conduction velocity was observed in diabetic rats one to 2 weeks after STZ injection, this normal functioning might be sustained by a robust ventricular conduction reserve. However, under challenging conditions like elevated potassium or ischemia, diabetic ventricles showed larger conduction times compared to controls (Nygren et al., 2007; Rahnema et al., 2011). Furthermore, overt impaired conduction velocity may develop as diabetes progresses. In this sense, long-term type 1 diabetic rabbits without cardiac fibrosis had decreased conduction already in basal conditions. Again, stressing the heart with hypo or hyperkalemia further slowed the velocity. A mechanism responsible might be the diabetes-induced reduction in the cardiac sodium current, as reported by electrophysiological techniques and mathematical modeling (Stables et al., 2014).

### Atrial Fibrillation

Diabetes is an independent risk factor for atrial fibrillation (AF) and diabetic patients have one-third greater risk of incidence of AF compared with unaffected individuals (Huxley et al., 2012). Although the underlying pathophysiological mechanisms are not completely understood, diabetes-induced atrial remodeling shares some mechanisms with diabetic cardiomyopathy. Atrial autonomic dysregulation, oxidative stress, fluctuations of glucose levels and structural and electrical remodeling contribute to the development of arrhythmia (Goudis et al., 2015; Wang et al., 2019). For instance, the above mentioned diabetes-induced differential expression of conexins, that affects action potential conduction, might contribute to the development of atrial fibrillation (Watanabe et al., 2012). Regarding the electrical remodeling, diabetic Zucker obese rats with higher susceptibility to AF showed prolonged atrial action potential duration due to a reduction of the ultrarapid delayed rectifier and transient outward repolarizing currents (I_kur_ and I_to_) along with a reduction of the corresponding channel forming proteins (Fu et al., 2019).

### Prolonged QT Interval Duration

**FIGURE 2:**
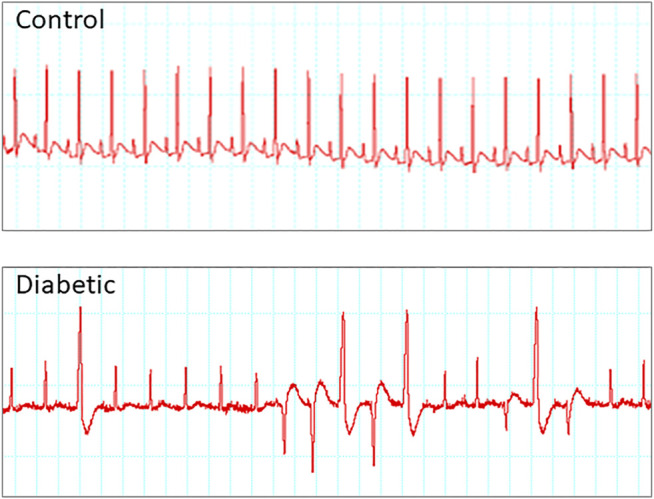
Diabetes is a substrate for developing arrhythmia. Example of electrocardiograms recorded from control and type 2 diabetic rats upon stimulation with caffeine plus dobutamine. Only diabetic animals develop arrhythmia under cardiac challenge.

The most-studied electrical alteration of the diabetic myocardium is prolonged ventricular repolarization. Diabetes-induced lengthening of QT and QTc intervals associates with higher risk of developing ventricular arrhythmias and sudden cardiac death (Figure 2). Prolonged QTc was first reported in individuals with diabetic neuropathy (Bellavere et al., 1988; Chambers et al., 1990; Jermendy et al., 1990; Ewing et al., 1991) and later, it was also observed in newly diagnosed type 2 diabetic patients with no apparent complications (Naas et al., 1998). It is difficult to estimate the prevalence of longer QTc among diabetics. The different characteristics of the cohorts in each study and the formula used to adjust the QT for heart rate (Bazett, Fridericia, Hodges, others) lead to heterogeneous results, with prevalence estimates ranging from 30 to 66% (Veglio et al., 2002; Kumar et al., 2004; Li et al., 2012b; Cox et al., 2014; Lu et al., 2017). In these studies, recruited participants usually exhibited diabetes with several years of duration and therefore received antidiabetic medication. Metformin is the first choice to start glycemic control and many patients require additional drugs over time (Ferrannini and DeFronzo, 2015). Most prevalence studies do not differentiate subjects on monotherapy from those on combined therapy, but the fact is that antidiabetic medication does not seem to be efficient to restore normal QTc values.

Another parameter that analyzes the repolarization is the QT dispersion or QTd, which reflects the difference between the longer and the shorter QT interval duration in the 12 leads electrocardiogram. An increase in the QTd is typically associated to an increased predisposition to ventricular arrhythmias. Diabetic patients show increased QTd and heart-rate corrected QT dispersion (QTdc) compared to non-diabetic subjects in the absence of other cardiovascular pathologies. Although the prevalence of severe prolonged QTc (>500 ms) and QTd (>80 ms) is low (Ninkovic et al., 2016), the presence of concomitant cardiovascular abnormalities such as left ventricular hypertrophy or hypertension increased the QT dispersion in diabetes mellitus (Cardoso et al., 2001).

## ION Channels and Currents in the Diabetic Myocardium

**FIGURE 3:**
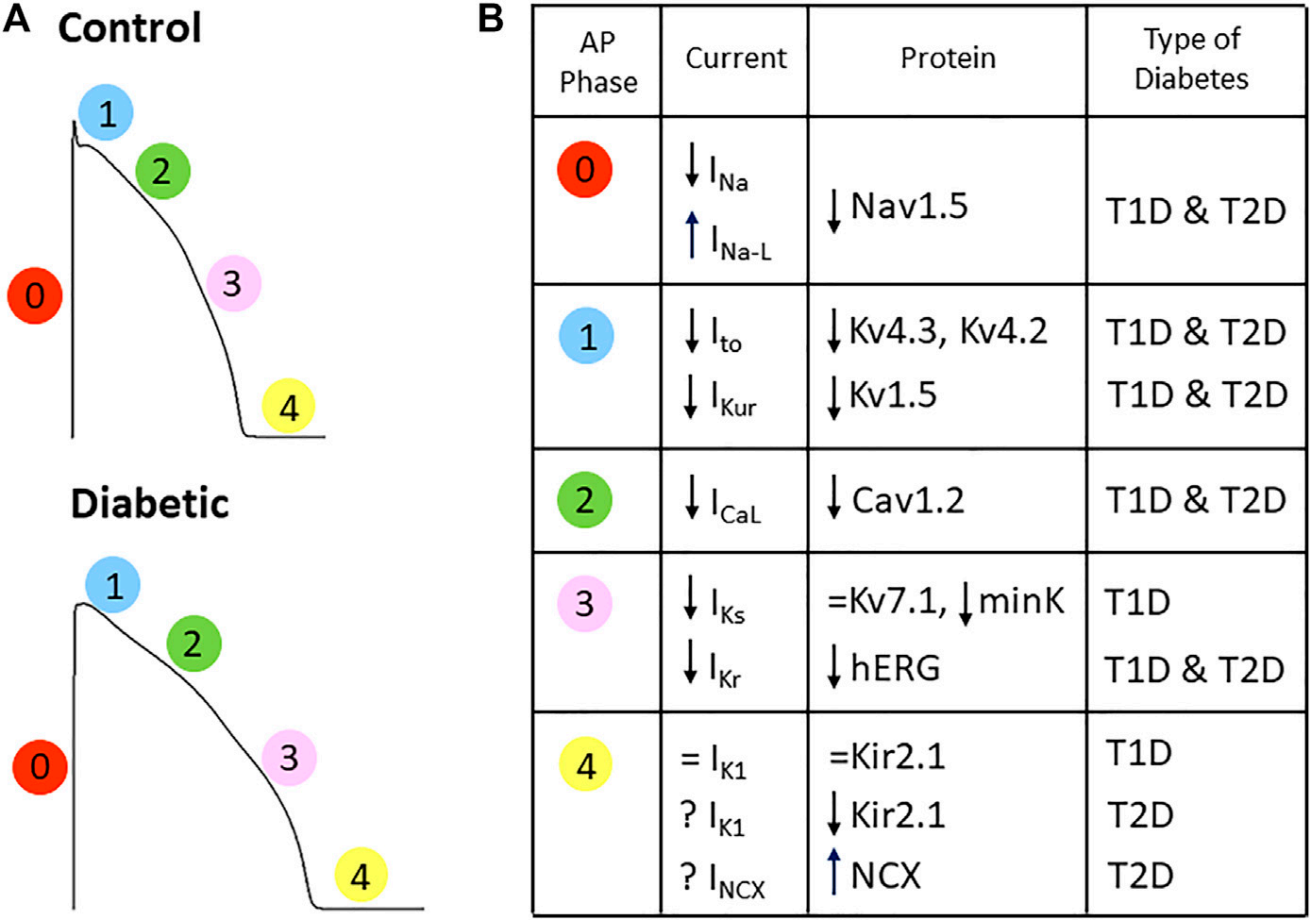
Diabetes affects the depolarizing and repolarizing currents responsible for the cardiac action potential. **(A)** Action potential depictions of a control and a diabetic heart (numbers indicate the phase of the AP) showing that diabetes changes action potential shape and prolongs its duration. **(B)** Summary of the alterations in the cardiac ion currents and the respective channel-forming proteins obtained in animal models of TD1 and T2D. See text for references.

The basic electrical activity of the cardiac cells is an action potential consisting of a rapid depolarization phase followed by a repolarization phase with a plateau. In 1983, Fein et al. described for the first time a lengthening of the cardiac action potential duration (APD) in a rat model of T1D (Fein et al., 1983), an effect consistently confirmed in rodent and non-rodent models (Magyar et al., 1992; Casis et al., 2000; Lengyel et al., 2007; Torres-Jacome et al., 2013). Thus, the APD observed in animal models of diabetes correlates with the prolonged QTc duration and increased QTd found in diabetic patients. In addition, in ventricular myocytes isolated from diabetic rats APD prolongation was not homogeneous throughout the heart, and the effect was more pronounced in the endocardium than in the epicardium (Casis et al., 2000). Prolonged APD results from diabetes-induced alterations in the expression and behavior of several ion channels that conduct depolarizing (sodium and calcium) and repolarizing (potassium) currents (summarized in Figure 3).

### Alterations in the Sodium Current

The protein Nav1.5, encoded by the SCN5A gene, is the cardiac isoform of the voltage-gated sodium channel, which is responsible for carrying the sodium current, or I_Na_. Diabetic rabbits displayed a reduction in the density of the cardiac I_Na_ although the Nav1.5 protein levels were not significantly reduced (Stables et al., 2014). However, in type 1 diabetic rats, as well as in culture cells exposed to hyperglycemic conditions, Yu et al. reported a reduced amount of Nav1.5 channels in the membrane. The excessive O-linked GlcNAcylation led to abnormal Nav1.5 aggregation in the cytoplasm and defective trafficking of the channel to the membrane (Yu et al., 2018). Nav1.5 channels open during the depolarization phase and then rapidly inactivate. However, some channels might reopen creating a persistent or late sodium current or I_Na,L_ that interferes with the repolarization (Zaza and Rocchetti, 2013). If the contribution of the late component of the I_Na_ increases, AP duration can prolong excessively increasing the risk of arrhythmia. This might happen in diabetes; in fact, increased I_Na,L_ has been found in type 1 and type 2 (db/db) mice with prolonged QTc (Lu et al., 2013). Moreover, diabetic mice treated with the I_Na,L_ inhibitor GS967 were more resistant to develop atrial fibrillation under an arrhythmia-inducing protocol than untreated diabetic animals (Jin et al., 2019). In addition, cultured cells incubated in high glucose conditions showed an increase in the late sodium current (Fouda et al., 2020). Different signaling pathways, metabolites and mechanisms regulate the late sodium current (Horváth et al., 2020). In diabetic mice, attenuated insulin/PI3K/Akt signaling increases I_Na,L_, contributing to the subsequent APD and QTc prolongations (Lu et al., 2013).

### Calcium Current and Calcium Handling

The L-type calcium current or I_Ca-L_ is a depolarizing current active during the phase of repolarization of the action potential and is the current responsible for the maintenance of the plateau phase. Regarding the I_Ca-L_, experiments with animal models have yielded confusing results. Some groups found no effects upon the current amplitude and the biophysical behavior of the calcium channel (Jourdon and Feuvray, 1993; Tsuchida et al., 1994; Lengyel et al., 2007; Lengyel et al., 2008) in type 1 diabetic models. On the contrary, other groups described a reduction in the current amplitude and a slowing in the current inactivation kinetics (Horackova and Murphy, 1988; Wang et al., 1995; Chattou et al., 1999). Finally, some groups reported a reduction of I_Ca-L_ but no effect on the channel behavior. In this sense, the reduction in the cardiac I_Ca-L_ density correlated with a decreased expression of its channel-forming protein Cav1.2 in Akita mice, a genetic model of T1D with defective insulin production, as well as in models of T2D like the db/db obese mice and Zucker obese rats (Pereira et al., 2006; Lu et al., 2007; Fu et al., 2019). Electrophysiological recordings of single-channel activity showed that the biophysical properties of the calcium channel were similar in control and diabetic animals (Pereira et al., 2006).

The reason behind these discrepant results is not clear. It might be related with methodological aspects in current recordings, such as the patch-clamp configuration or the charge carrier used in the experiment (Ca^2+^ or Ba^2+^). There could also be intrinsic differences in the diabetic models, for instance, induced *vs.* genetic. The experimental conditions for current recordings are also crucial, since the amplitude of the L-type calcium current is very sensitive to the intracellular calcium content. In physiological conditions, L-type calcium channels inactivate *via* Ca^2+^-dependent inactivation, mainly by the calcium released from the sarcoplasmic reticulum (Pelzer et al., 1990; Sham, 1997; Kubalová, 2003). Thus, if intracellular Ca^2+^ is buffered during the current recording, differences in current behavior between control and diabetic cardiomyocytes might not be observed. However, experimental conditions that preserve the intracellular Ca^2+^ can differentially affect the behavior of the I_Ca-L_ in control and diabetic cells. In fact, intracellular calcium handling is altered in the diabetic myocardium (Allo et al., 1991), showing a characteristic reduced systolic Ca^2+^ and an augmented diastolic Ca^2+^.

Diabetes induces changes in several proteins involved in calcium handling, what leads to an increase in the cytosolic (Ca^2+^). Diabetes prolongs the duration of both the contraction and the relaxation phases of the cardiac cycle (Regan et al., 1974; Feuvray et al., 1979; Fein et al., 1980). In the diabetic heart, Ca^2+^ release from the sarcoplasmic reticulum during systole is depressed, mainly due to a downregulation of Ca^2+^-releasing proteins at the SR membrane. In this sense, mRNA and protein levels of ryanodine receptors (RYRs) are reduced in diabetes and become fully restored after insulin supplementation (Teshima et al., 2000; Netticadan et al., 2001). However, more characteristic of the diabetic myocardium is the diastolic calcium overload, which is increased about three fold (Allo et al., 1991). Diabetes increases the opening probability of RYRs and leads to diastolic Ca^2+^ leak to the cytoplasm (Singh et al., 2018). In addition, calcium reuptake to the SR is also compromised because diabetic cardiomyopathy reduces the expression of the sarcoplasmic reticulum Ca^2+^-ATPase (SERCA2) as well as its apparent affinity for Ca^2+^ (Teshima et al., 2000; Netticadan et al., 2001), (Zhong et al., 2001; Abe et al., 2002; Razeghi et al., 2002). As a result, diabetic cardiomyocytes have higher diastolic calcium levels caused by a markedly impaired calcium uptake (Penpargkul et al., 1981; Horackova and Murphy, 1988; Fein et al., 1990; Golfman et al., 1996). Thus, diabetic cardiomyocytes have less calcium available for the contraction during systole together with an excess of diastolic calcium that impairs relaxation during diastole.

### The Repolarizing Potassium Currents

Several potassium currents determine the action potential repolarization phase. In the human heart these are the transient outward or I_to_, the ultrarapid delayed rectifier or I_Kur_, the rapid delayed rectifier or I_Kr_, the slow delayed rectifier or I_Ks_, and the inward rectifier or I_K1_ (Chiamvimonvat et al., 2017). Diabetes does not change the inward rectifier, the current that contributes to the final phase of the repolarization (Magyar et al., 1992; Casis et al., 2000).

On the contrary, I_to_, which is the dominant ventricular repolarizing current in rodents, is highly affected by T1D. A reduction in the expression of the channel-forming proteins Kv4.3 and Kv4.2 and the accessory subunit KChIP2 (Qin et al., 2001; Lengyel et al., 2007; Torres-Jacome et al., 2013) caused the reduction in the amplitude of I_to_ (Magyar et al., 1992; Shimoni et al., 1994; Xu et al., 1996; Casis et al., 2000). In addition, diabetes accelerated I_to_ current inactivation (Magyar et al., 1992; Casis et al., 2000) due to a reduction in the Kv4.3 channel phosphorylation by CaMKII (Gallego et al., 2008). This contributed to the reduction of the total current and the lengthening of the action potential duration. Regarding I_Kur_, diabetes reduced the expression of the pore forming protein Kv1.5, thus inhibiting the current (Casis et al., 2000; Torres-Jacome et al., 2013).

The delayed rectifiers are virtually absent in rodents; therefore, they have been analyzed mostly in type 1 diabetic rabbits and dogs. The amplitude of the slow delayed rectifier I_Ks_ was reduced (Lengyel et al., 2007; Lengyel et al., 2008) which correlated with the reduced expression levels of the accessory subunit mink, whereas the pore-forming subunit Kv7.1 was not or little affected (Lengyel et al., 2007; Zhang et al., 2007). Regarding the rapid delayed rectifier, most of the studies reported no effect of diabetes on I_Kr_ amplitude and properties (Lengyel et al., 2007; Lengyel et al., 2008; Torres-Jacome et al., 2013). However*,* experiments made in rabbits with long diabetes duration showed significant reduction of both the I_Kr_ amplitude and the expression of its pore-forming protein ERG (Zhang et al., 2006; Zhang et al., 2007).

A combination of factors can lead to the reduced electrical activity and expression of cardiac potassium channels. In type 1 diabetic animals, insulin treatment restored some but not all the altered currents (Zhang et al., 2006; Lengyel et al., 2007). The impaired metabolic status of the diabetic cells might also affect protein synthesis. For instance, *in vitro* activation of the AMP-dependent protein kinase reduced several K^+^ repolarizing currents in a similar fashion than diabetes (Torres-Jacome et al., 2013). On the other hand, diabetes induced a sterile inflammation that increased the IL-1b release from cardiac macrophages and led to the reduction of the I_to_ (Monnerat et al., 2016).

The literature regarding cardiac electrical remodeling in T2D, however, is very limited. A few studies reported a prolongation of APD and a reduction of the amplitude of the ventricular I_to_ current in the genetic models WBN/Kob rats (Tsuchida et al., 1994; Tsuchida and Watajima, 1997), leptin-receptor deficient homozygous db/db mice (Shimoni et al., 2004) or the Otsuka-Long-Evans-Tokushima Fatty rats (Sato et al., 2014). Similarly, I_to_, I_Kur_ and I_Ca-L_ currents, as well as their corresponding channel-forming proteins, were reduced in the atria of Zucker diabetic fatty rats (Fu et al., 2019). Regarding humans, a study in elderly type 2 diabetic patients (Ashrafi et al., 2017) showed a reduction of the mRNAs encoding for hERG and Kir channels responsible for I_Kr_ and I_K1_, and an increase of the Na^+^/Ca^2+^ exchanger (NCX) expression. Although channel protein levels and current recordings were not assessed and the study was performed in few, aged and poly-medicated patients, this is the first work that directly compares the expression of cardiac channels between diabetic and non-diabetic humans (Hancox, 2017).

## Safety Assessment of Antidiabetic Drugs

### Proarrhythmic Safety of New Drugs

Like diabetes, other disorders cause metabolic, endocrine, immune or autonomic disturbances, providing an environment that impairs cardiac ion channel function. Moreover, some drugs can directly inhibit and in some cases activate ion channels, affecting the overall electric response. Therefore, the proarrhythmic propensity of drugs is a matter of concern. Between 1989 and 2003 several drugs were associated with ventricular arrhythmia, mainly *torsade de pointes*, and were withdrawn from the market [reviewed in Turner et al., 2018]. As a result, in 2005 the International Council for Harmonization (ICH) of Technical Requirements for Pharmaceuticals for Human Use released two guidelines for industry, the non-clinical S7B and the clinical E14, to evaluate potential proarrhythmic effects of new drugs.

Non-clinical testing strategy focused mainly on evaluating the effects of a drug on the hERG channel *in vitro*, and in electrocardiographic recordings to analyze ventricular repolarization (ICH S7B Guideline, 2005). The hERG channel encoded by the human ether-a-go-go-related gene conducts the I_Kr_, the main repolarizing current in humans. The blockade of hERG channel is responsible for most of the drug-induced QT prolongations and TdPs because the protein has a peculiar site that effectively accommodates the binding of drugs and makes it particularly susceptible to blockade (Sanguinetti and Tristani-Firouzi, 2006). However, not all the hERG-channel blockers prolong the QT interval or induce TdP. Some drugs might have additional effects on other channels that counteract the lengthening of the repolarization, resulting in normal action potential and QT interval duration. Furthermore, QTc prolongation does not necessarily trigger TdP yet may discontinue compounds from development. Since 2013, the Comprehensive *in vitro* Proarrhythmia Assay (CiPA) initiative works in a new paradigm for assessment of TdP proarrhythmic risk of new drugs that is not focused exclusively in hERG blockage and QT prolongation (Sager et al., 2014). Instead, the CiPA initiative includes: the *in vitro* assessment of drug effects on multiple ion channels; the prediction of proarrhythmic risk using *in silico* models; the *in vitro* confirmation of proarrhythmicity in human stem cell derived ventricular cardiomyocytes; and electrocardiograms in phase 1 clinical trials (Vicente et al., 2018).

On the other hand, the clinical evaluation of potential proarrhythmic effects consists on the “Thorough QT/QTc” or TQT Study, usually performed before phase 3 clinical development to detect whether the drug has a threshold pharmacologic effect on prolonging repolarization. In the TQT studies, healthy voluntaries receive a negative control (placebo); a positive control (a drug that prolongs the QTc); the drug under development at the maximum recommended therapeutic dose; and the drug at supratherapeutic dose to explore the “worst-case scenario”, for instance, in an impaired clearance of the drug. The TQT study is negative if the drug does not prolong the mean QT/QTc interval more than 5 ms, which is considered the threshold of regulatory concern (ICH E14 Guideline, 2015). However, the TQT study is resource intensive and scientists and regulatory agencies are discussing alternatives that are more effective, such as the intensive assessment of ECG parameters in the first-in-human study (Darpo et al., 2014; ICH E14 Guideline, 2015).

### Proarrhythmic Safety of Antidiabetic Drugs

Since metformin, insulin, sulfonylureas or thiazolidinedione were marketed years before the regulations about arrhythmic safety, TQT studies did not evaluate these classic antidiabetics. On the contrary, newer drugs approved after the ICH E14 guideline, such as semaglutide, have been examined in TQT studies (Demmel et al., 2018). However, evaluation of glucose lowering drugs is very challenging since, for instance, changes in blood glucose concentrations *per se* may correlate with prolonged QTc (Suys et al., 2006). The Cardiac Safety Research Consortium discussed these major confounding factors (Heller et al., 2015). Variations in glucose, insulin and potassium levels, fasted vs. fed state and the activation of the autonomous nervous system may all affect ventricular repolarization, as well as other ECG parameters like RR interval duration and the T-wave morphology. This complicates the design and interpretation of TQT studies for assessing antidiabetic compounds.

### Glucose-Lowering Treatment for Patients at Cardiovascular Risk

On the other hand, although the incidence of cardiovascular disease (CVD) has declined in both adults with and without diabetes over the last few decades, diabetic patients still have 2-fold greater risk of CVD compared with the non-diabetic population (Fox et al., 2004; Preis et al., 2009). In addition, studies of the agonist of the peroxisome proliferator-activated receptor families alpha and gamma muraglitazar, an agonist of the peroxisome proliferator-activated receptor (PPAR), and the thiazolidinedione rosiglitazone, concluded an increase in the risk of death and in the incidence of major adverse cardiovascular events compared to standard therapy (Nissen et al., 2005; Nissen and Wolski, 2007). These concerning results precipitated that, in 2008, the FDA issued guidance for industry aimed to ensure that new antidiabetic therapies for T2D do not increase the CVD risk. As a result, during pharmacological development of new antidiabetic drugs, Cardiovascular Outcome Trials (CVOTs) must be performed on selected type 2 diabetic patients at higher risk of cardiovascular events (Food and Drug Administration, 2008). Primary outcome in CVOTs is typically a composite of death of cardiovascular cause, non-fatal myocardial infarction and non-fatal stroke. Secondary outcomes may include hospitalization for heart failure, acute coronary syndrome and revascularization (Schnell et al., 2016). Interestingly, diabetes is the only pathology where routine CVOTs in the absence of safety signals are mandatory.

As with the TQTs, CVOTs were not performed for the old antidiabetic drugs. Three groups of new drugs: dipeptidyl peptidase 4 (DPP-4) inhibitors, sodium-glucose cotransporter 2 (SGLT2) inhibitors and glucagon-like peptide 1 (GLP-1) receptor agonists have undertaken CVOTs. Detailed summaries of completed and ongoing studies have been recently published (Cefalu et al., 2018; Schnell et al., 2019; Schnell et al., 2020). Although some differences between drugs exist, CVOTs confirm that the tested drugs fulfill the FDA requirements because they are not associated with an unacceptable increase in cardiovascular risk*;* therefore, all of them are safe. The GLP1R agonist liraglutide and the SGLT2 inhibitors empagliflozin and, to a lower extent, canagliflozin, have yielded very positive results. Consequently, the American Diabetes Association recommended incorporating these drugs to the standard therapy in T2D patients with established atherosclerotic cardiovascular disease (Davies et al., 2018).

Furthermore, very recent studies have focused on the potential protective effect of SGLT2 inhibitors against developing arrhythmias. In newly diagnosed diabetic patients, SGTL2 inhibitors associates with a reduced risk of new onset arrhythmia (Chen et al., 2020). Similarly, recent meta-analysis examining arrhythmia outcomes, like atrial fibrillation and ventricular tachycardia, found that SGLT2 inhibitors treatment reduces the risk of cardiac arrhythmias in diabetic patients (Li et al., 2021). However, in most trials, information regarding pre-existing arrhythmia and anti-arrhythmic therapy were not available. More research will be required to determine the best treatment for those arrhythmic patients who develop diabetes. Currently, the ADA recommends the use of either an SGLT2 inhibitor or a GLP-1 receptor agonist to reduce cardiovascular risk because this treatment is appropriate for many patients (ADA, 2021). Although both SGLT2i and GLP-1R agonist could be used in patients with atherosclerotic cardiovascular disease, SGLT2 inhibitors are recommended in patients with heart failure or kidney disease (Dardano et al., 2020).

Among the proposed cardioprotective mechanisms are lowering blood pressure, which may lower cardiac afterload; improving cardiac metabolism increasing energy production; reducing inflammation and cytokine release; preventing adverse cardiac remodeling; reducing sympathetic nerve activity; improving renal function; improving mitochondrial dysfunction and improving ionic dyshomeostasis. Thus, the underlying cardioprotective mechanisms of SGLT2 inhibitors and GLP-1 receptor agonists remain unclear and might involve complementary systemic and direct cardiac effects (recently reviewed in Lahnwong et al., 2018; Dardano et al., 2020; Lopaschuk and Verma, 2020).

Regarding ionic dyshomeostasis and arrhythmic risk, (Na^+^) was elevated in the diabetic myocardium, directly increasing the risk of sudden arrhythmic death (Lambert et al., 2015). Empagliflocin inhibits the Na^+^/H^+^ exchanger and reduces the sodium and calcium concentration (Baartscheer et al., 2017). Another SGLT2 inhibitor, dapagliflozin, reduced the membrane expression of the Na^+^/H^+^ exchanger, the Na^+^/Ca^2+^ exchanger and the L-type calcium channel in db/db mice (Arow et al., 2020). The resulting reduction of the cytoplasmic (Na^+^) and (Ca^2+^) might reduce the risk of developing potentially lethal arrhythmias. Very interestingly, in insulin resistant rats dapagliflozin-treatment improved cardiac repolarization. Daplagliflozin supressed QT interval and AP prolongation, mainly by restoring the depressed potassium currents I_to_ and I_K1_, but also by reducing I_Na_ (Durak et al., 2018).

## Conclusion and Future Prespectives

Both T1D and T2D induce a cardiac remodeling that leads to mechanical dysfunction and/or cardiac arrhythmias. Experiments with animal models have consistently shown that diabetes alters the expression and regulation of cardiac ion channels and transporters, which impairs impulse generation, conduction, excitation-contraction coupling and myocyte contractility.

Prolonged QTc interval duration persists in a number of treated diabetic patients, indicating that strict glycemic control is not sufficient to normalize the electrophysiological disturbances. Available glucose-lowering drugs that improve cardiovascular prognosis are crucial in the management of type 2 diabetic patients with established cardiovascular disease or at high cardiovascular risk. Further studies are needed to elucidate if cardioprotection includes electrical remodeling and prolonged repolarization. This could be of particular interest for patients with diabetes-associated complications that may increase the risk of arrhythmia.
